# Chemical approaches to targeted protein degradation through modulation of the ubiquitin–proteasome pathway

**DOI:** 10.1042/BCJ20160762

**Published:** 2017-03-15

**Authors:** Ian Collins, Hannah Wang, John J. Caldwell, Raj Chopra

**Affiliations:** Cancer Research UK Cancer Therapeutics Unit, The Institute of Cancer Research, London SM2 5NG, U.K.

**Keywords:** chemical biology, chemical tools, ubiquitin ligases, ubiquitin–proteasome system

## Abstract

Manipulation of the ubiquitin–proteasome system to achieve targeted degradation of proteins within cells using chemical tools and drugs has the potential to transform pharmacological and therapeutic approaches in cancer and other diseases. An increased understanding of the molecular mechanism of thalidomide and its analogues following their clinical use has unlocked small-molecule modulation of the substrate specificity of the E3 ligase cereblon (CRBN), which in turn has resulted in the advancement of new immunomodulatory drugs (IMiDs) into the clinic. The degradation of multiple context-specific proteins by these pleiotropic small molecules provides a means to uncover new cell biology and to generate future drug molecules against currently undruggable targets. In parallel, the development of larger bifunctional molecules that bring together highly specific protein targets in complexes with CRBN, von Hippel–Lindau, or other E3 ligases to promote ubiquitin-dependent degradation has progressed to generate selective chemical compounds with potent effects in cells and *in vivo* models, providing valuable tools for biological target validation and with future potential for therapeutic use. In this review, we survey recent breakthroughs achieved in these two complementary methods and the discovery of new modes of direct and indirect engagement of target proteins with the proteasome. We discuss the experimental characterisation that validates the use of molecules that promote protein degradation as chemical tools, the preclinical and clinical examples disclosed to date, and the future prospects for this exciting area of chemical biology.

## Introduction

The process by which proteins are systematically degraded in a timely manner represents a fundamental mechanism for maintaining protein and cellular homeostasis. Protein homeostasis is mainly regulated by the ubiquitin–proteasome pathway, which was initially discovered by studying the degradation of denatured globin in reticulocyte lysates by the seminal work of Ciehanover, Hershko, Rose and colleagues [11,25]. Their work showed that proteins were targeted for degradation in an ATP-dependent manner by covalent conjugation of multiple molecules of an ATP-dependent proteolytic factor, APF-1, later identified as ubiquitin. Subsequent work showed that ubiquitin-mediated protein degradation occurs in a stepwise manner through an enzymatic cascade starting with activation of ubiquitin by the E1 ubiquitin ligase enzymes (UBEs), ubiquitin-like modifier-activating enzymes (UBAs) 1 and 6. Activated ubiquitin is then transferred to a ubiquitin-conjugating enzyme E2, of which there are ∼30 examples. Subsequently, the members of the E3 ligase family (consisting of ∼600 enzymes) transfer ubiquitin to the target protein substrate [4].

Polyubiquitination occurs via the creation of isopeptide bonds between the C-terminal glycine of ubiquitin and the N-terminal methionine or one of several lysine residues in the substrate ubiquitin. Attachment of multiple ubiquitin molecules through conjugation to lysine-48 residues is associated with protein degradation via the proteasome. On the other hand, polyubiquitination via lysine-63 is associated with creating scaffolds for cell signalling and other critical biological processes [32]. Ubiquitination is reversed by ∼100 deubiquitinase enzymes (DUBs) that are proteases consisting of five subfamilies: ubiquitin-specific proteases (USP), ubiquitin carboxyl-terminal hydrolases (UCH), ovarian tumour-like (OTUs), Machado-Joseph disease (MJD) protein domain proteases and the JAMM (JAB1/MPN/MOV34) metalloprotease family [49].

**Figure 1.**
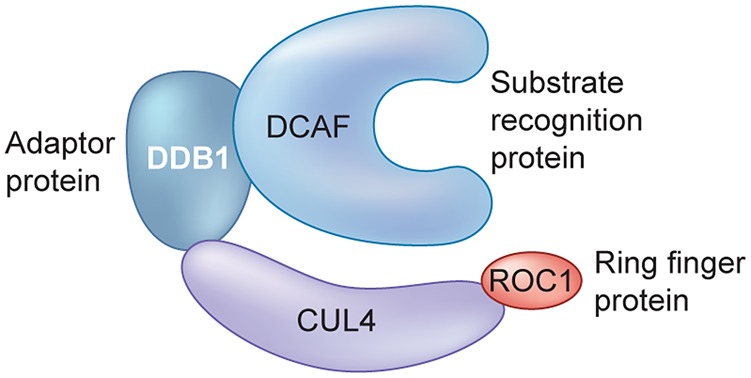
An example of an E3 ligase complex. Components of the CUL-RING ligase 4 (CRL4). CUL4, Cullin-RING ligase 4; DCAF, DDB1- and CUL4-associated factor; DDB1, DNA damage-binding protein 1; ROC1, RING-box protein 1.

The E3 ligases consist of four different classes of enzymes [51]. These include the really interesting new gene (RING) domains that recognise target proteins and mediate transfer of ubiquitin from E2; the homologous to the E6-AP carboxyl terminus (HECT) domains, which transfer ubiquitin to their own cysteine residues and then onto the target substrate protein; the u/box E3 ligases that function like RING E3 ligases, having a RING-like domain but lacking the cysteine and histidine zinc co-ordination sites; and finally, the recently described RING-in-between-RING (RBR) family, which behaves as a hybrid between RING and HECT enzymes. The largest family of E3 ligases is the cullin (CUL)-RING ligases (CRLs), which play a role in many diverse cellular processes [62]. Most CRL E3 ligases are assembled in a modular form [63], where the CUL protein acts as a molecular scaffold that assembles the multisubunit CRL complexes. The functional complexes consist of four subunits — the CUL protein, which engages a receptor protein for substrate recognition, an adaptor protein, and a RING finger E3 ligase that binds to a ubiquitin-charged E2 that catalyses the transfer of ubiquitin to specific substrates. A major part of this review concerns the CRL4 sub-family, and Figure 1 shows the key components of an important CUL4 complex. Cereblon (CRBN) is the substrate receptor for this CRL4 complex, with DNA damage-binding protein 1 (DDB1) serving as the adapter protein and CUL4 as the scaffold. Substrate receptors interacting with DDB1 are also known as DDB1- and CUL4-associated factors (DCAFs) [1]. There are a large number of DCAFs, including CRBN, that are involved in the targeting of a wide range of substrates for ubiquitination, resulting in the regulation of a broad range of essential cellular processes including DNA repair, DNA replication, and chromatin remodelling [38]. Roc1 (also called Rbx1) is the RING finger E3 ligase that is part of the CRL4^CRBN^ complex.

Given the critical role of the ubiquitin–proteasome system in cellular physiology and its dysregulation in many diseases including cancer, neurodegenerative diseases, infections, metabolic disorders, and inflammation, there is a great deal of recent endeavour to target components of this system for drug discovery and development. Indeed, it has been suggested that the current efforts mirror the enthusiasm for targeting the kinases in the late 1990s and 2000s [13]. The aim of this review is to critically evaluate the current work and convey the excitement for using chemical approaches for targeting protein degradation through modulation of the ubiquitin–proteasome pathway. A key early exemplar of success in this area was the development of proteasome inhibitors such as bortezimib and carfilzomib [50]. Prior to that targeting of neddylation, which is related to the ubiquitin–proteasome pathway, has also shown promising results [17]. There is also a great deal of effort to target the DUBs [49].

**Figure 2.**
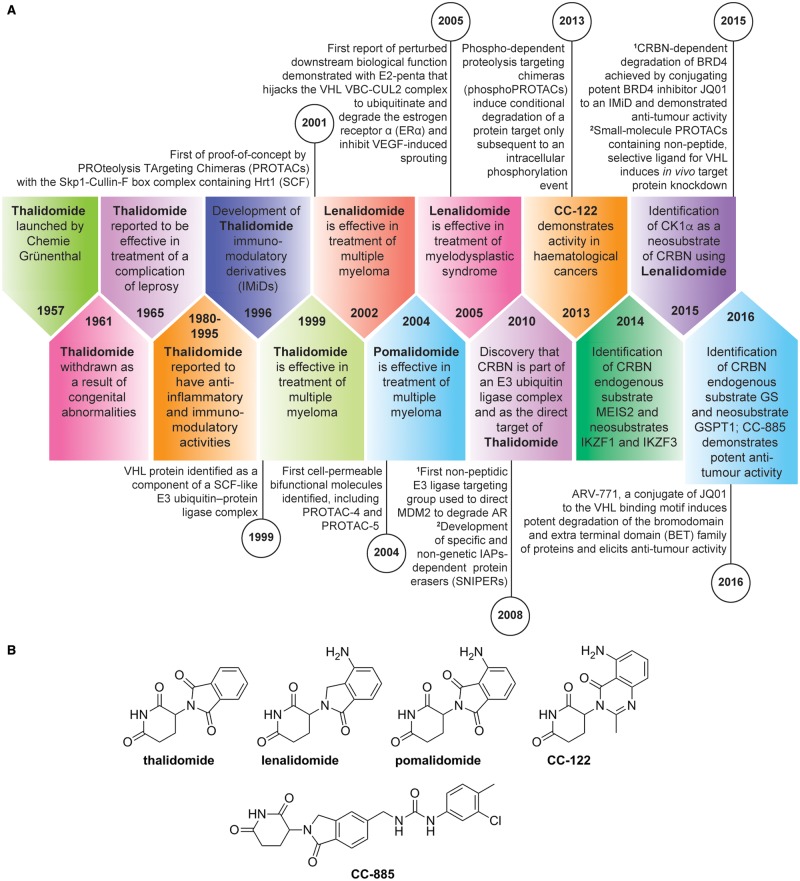
Timelines for the exploitation of E3 ligases for drug discovery and chemical biology. (**A**) Timeline (block arrows) of the development of immunomodulatory drugs (IMiDs), the discovery of CRBN, and its substrates. A timeline (circles) of key steps in the development of bifunctional molecules hijacking E3 ligases described in this review is shown in parallel. (**B**) Structures of published IMiDs.

In this review, we will focus on two complementary approaches to achieve targeted protein degradation (Figure 2A). First, we consider how small molecules can be used to directly modulate the CRL4^CRBN^ complex to change its specificities for substrate binding and thus redirect the spectrum of target degradation. This has already led to small-molecule drugs effective in haematological cancers [76]. Second, we survey the substantial recent data and examples demonstrating how CRBN and other E3 ligases may be hijacked by bifunctional molecules designed to deliver specific target proteins to the complex for ubiquitination and degradation [71,16,46,74,36]. This approach has already delivered chemical tools that allow target depletion in cells or *in vivo* to be studied using pharmacological agents as an alternative to RNA interference or other genetic methods. Furthermore, we discuss how some of these approaches may enable the drugging of hitherto difficult to drug proteins.

## Direct modulation of CRBN E3 ligase substrate specificity by small molecules

In the early 1960s, thalidomide was infamously withdrawn from the market after reports of severe birth deformities in infants born to women who took the anti-morning sickness drug during pregnancy. Since then thalidomide has been demonstrated to be effective in treating a complication of leprosy and also multiple myeloma (MM), owing to its antiangiogenic, immunomodulatory, and anti-inflammatory properties [47,60,69,3]. However, thalidomide's molecular target remained unknown until 2010 when Hiroshi Handa's laboratory in Tokyo discovered through a series of affinity purification assays that thalidomide directly binds to CRBN and inhibits its ubiquitination [28]. Notably, Ito et al. [28] showed that the phthalimide portion of the thalidomide structure did not bind to CRBN and that CRBN is the protein target of thalidomide responsible for thalidomide-mediated teratogenesis in zebrafish.

**Figure 3.**
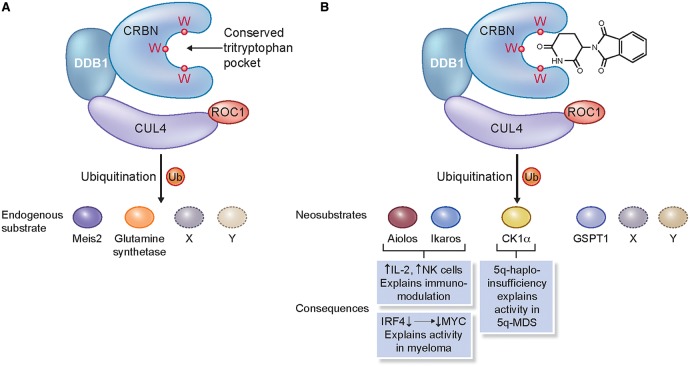
The consequences of perturbation of CUL4^CRBN^ E3 ligase function. (**A**) The role of CRL4^CRBN^ in mediating protein homeostasis of endogenous substrates (Meis2 and glutamine synthetase); the conserved tritryptophan pocket that binds IMiDs is highlighted. (**B**) Known protein substrates of consequence (for example, Aiolos, Ikaros, CK1α, and GSPT1) whose rate of degradation is altered in response to the binding of thalidomide to the tritryptophan pocket of CRL4^CRBN^; as yet undiscovered substrates are denoted by X and Y.

The revival of thalidomide's clinical utility has since led to the development of more potent and less toxic analogues known as immunomodulatory drugs (IMiDs), such as lenalidomide and pomalidomide (Figure 2B). CRBN was also identified as the target of lenalidomide and pomalidomide, and is responsible for the immunomodulatory and antiproliferative activities of these agents in MM [44]. It was hypothesised that IMiDs alter the abundance, localisation, and activity of CRL^CRBN^ E3 ligase substrates (Figure 3A,B) [78,44]. Three studies in 2014 by the Ebert, Kaelin, and Chopra groups [22,33,45] showed that these changes arise from the ability of IMiDs to alter CRBN's E3 ligase substrate preference, resulting in the ubiquitination and degradation of the transcription factors Ikaros (IKZF1) and Aiolos (IKZF3). The data from these and subsequent studies also demonstrated that Ikaros and Aiolos degradation was dependent on the presence of IMiDs and therefore represents drug-induced neomorphic activity, with Ikaros and Aiolos identified as neosubstrates of the CRBN E3 ligase complex [39]. It was subsequently shown that, in MM, proteasomal degradation of Ikaros and Aiolos resulted in the down-regulation of c-MYC followed by a decrease in interferon regulatory factor 4 (IRF4) expression, and that this was associated with growth inhibition and apoptosis. These results suggested a functional link between Ikaros and Aiolos, and the pathological deregulation of c-MYC and IRF4 in MM, which had hitherto not been described [6,24]. Furthermore, the degradation of Aiolos and Ikaros in T-cells [22] explained, at least in part, the activation of the immune system seen in patients receiving IMiD compounds [21]. Both Aiolos and Ikaros were shown to be repressors of T-cell function and their degradation results in interleukin 2 production and T-cell activation. Of note, lenalidomide and known existing thalidomide analogues do not affect Aiolos and c-MYC in epithelial tumours, suggesting that in this context Aiolos and c-MYC are regulated through different mechanisms from those in B-cell malignancies.

Recently, the enzyme casein kinase 1α (CK1α) was discovered as a target of ubiquitin-mediated degradation in the presence of lenalidomide. Treatment with lenalidomide resulted in almost complete loss of CK1α in primary human stem cells derived from patients with myelodysplastic syndrome [del (5q) MDS] [34]. The gene that encodes CK1α, CSNK1A1, is on the long arm of chromosome 5, and is haploinsufficient in del (5q) MDS. CK1α regulates the activity of multiple proteins; for example, it negatively regulates TP53, a tumour-suppressor protein [66]; therefore, degradation of CK1α by lenalidomide restores TP53 and leads to clonal extinction of the del (5q) clone. This demonstrates that thalidomide analogues that change E3 ligase substrate specificity have the ability to unearth novel biology and, furthermore, to modulate a hitherto difficult to drug pathway. In the present study, thalidomide, pomalidomide, and CC-122, a novel CRBN-binding molecule (Figure 2B), did not degrade CK1α, highlighting that even though these compounds have similar chemical structures, with a shared glutarimide ring, they can have both unique and common substrate-modifying specificities.

The difference in CRBN E3 ligase substrate specificity was recently explained by elucidation of the crystal structures of the DDB1–CRBN complex bound to thalidomide, lenalidomide, and pomalidomide, reported by both the Celgene and Thoma groups [10,19]. These structures establish that CRBN is a substrate receptor within the CRL4^CRBN^ complex that enantioselectively binds IMiD compounds. The glutarimide ring of thalidomide and its analogues binds into a hydrophobic tritrytophan pocket, termed the thalidomide-binding domain (TBD), which is evolutionarily conserved from *Archea* to higher mammals [5,34]. The phthalimide ring is exposed on the surface of the CRBN protein and alters the surface of the E3 ligase substrate receptor to enable interaction with new substrates. More recently, the Thoma group extended the work on CK1α by elucidating a highly informative 2.45 Å crystal structure of DDB1–CRBN bound to lenalidomide and CK1α [55]. In this structure, CRBN and lenalidomide jointly provide the binding interface for a CK1α β-hairpin loop located in the kinase N-lobe. Interestingly, the structure reveals that CK1α binding to CRL4^CRBN^ is strictly dependent on the presence of an IMiD [55]. Moreover, the presence of Gly40 in the β-hairpin loop is important for mediating the degradation of target substrates, implying that this residue may form part of the protein degradation motif (degron) recognised by the IMiD–CRBN complex. Furthermore, the binding of Ikaros to CRBN similarly requires the presence of an IMiD compound, and both protein substrates, Ikaros and CK1α, adopt a related binding mode. These important high-affinity protein–protein interactions, which are specifically induced by small molecules, will provide opportunities for future drug discovery, particularly for targeted protein degradation.

It is expected that the binding of IMiDs to CRBN will displace endogenous substrates, of which little is known, and promote the recruitment of neosubstrates (Figure 3A,B). Recently, the homeobox gene, MEIS2, was identified as an endogenous substrate of CRBN, whereby MEIS2 ubiquitination is inhibited by the IMiDs, resulting in increased protein levels. These data indicate that IMiDs modulate ubiquitination; in some instances, creating a neomorph for substrate degradation, as for Aiolos and Ikaros, and in others, competing out endogenous substrates, such as MEIS2, thereby leading to a decrease in their degradation [19]. More recently, glutamine synthetase (GS) has also been shown to be an endogenous substrate of CRL4^CRBN^ [53]. GS recognition by CRBN leads to its polyubiquitination by CRL4^CRBN^ in response to high glutamine levels. Contrary to the case of MEIS2, IMiDs enhance GS binding to CRBN. Furthermore, two lysine resides (K11 and K14) in the N-terminus of GS are acetylated by p300/CBP in response to high glutamine concentrations. These acetylation marks serve as a degron to allow CRBN-binding and CRL4^CRBN^-mediated ubiquitination. These data suggest that, so far, there does not appear to be an identifiable, universally conserved degron motif and that CRL4^CRBN^-mediated degradation of substrates is likely to be dependent on both the cellular context and metabolic state of the cell.

CC-122 and CC-885 are the most recently developed IMiDs (Figure 2B). CC-122 is a pleiotropic pathway modifier that also binds CRBN and promotes degradation of Aiolos and Ikaros in diffuse large B-cell lymphoma (DLBCL) T-cells in *in vitro* and *in vivo* models and in patients, resulting in both cell autonomous and immune-stimulatory effects. In DLBCL cell lines, both CC-122-induced degradation and short hairpin RNA-mediated knockdown of Aiolos and Ikaros correlate with increased transcription of interferon (IFN)-stimulated genes; this is independent of IFN-α, -β, and -γ production and/or secretion, and results in apoptosis in both activated B-cell (ABC) and germinal centre B-cell DLBCL cell lines [24]. This drug is now entering Phase II/III studies for poor-risk lymphoma patients.

CC-885 is the first IMiD to demonstrate potent antitumour activity in both haematological and epithelial cancers [48]. In addition to inducing CRBN-mediated degradation of Ikaros as seen with other thalidomide analogues, CC-885 promotes the degradation of the translation termination factor GSPT1, resulting in cytotoxicity. Lenalidomide and pomalidomide do not degrade GSPT1, possibly due to their lack of the extended urea moiety of CC-885 that enables additional interactions with CRBN and GSPT1. Intriguingly, structural studies of CRBN–DDB1 with CC-885 and GSPT1 revealed that CC-885 creates a hotspot on the CRBN surface for direct interaction with GSPT1 and this interaction is not determined by a peptide sequence but rather by the geometric arrangement of three hydrogen bond acceptors on the GSPT1 backbone and the precise position of its glycine residue.

It is clear that the ability of IMiDs to direct CRL4^CRBN^ to degrade several different proteins opens up the potential to discover new effects of protein degradation and target hitherto undruggable proteins and pathways. This approach will be even more powerful if we are able to predict and select which proteins are degraded. One avenue is to use sequence and structural homologies in putative CRL4^CRBN^ degrons to define potential substrate proteins and to design small-molecule CRL4^CRBN^ binders that can selectively direct the degradation of these proteins. This rational design approach complements the empirical phenotypic screening employed to discover the existing IMiDs. Currently, defined degron sequences are cell context-dependent and vary according to the IMiD, with small changes in chemical structure leading to altered substrate specificity. Thus, further understanding is required to inform such a rational degron-based approach.

## Hijacking E3 ligases for specific target degradation using bifunctional molecules

### Hijacking the von Hippel–Lindau E3 ligase

**Figure 4.**
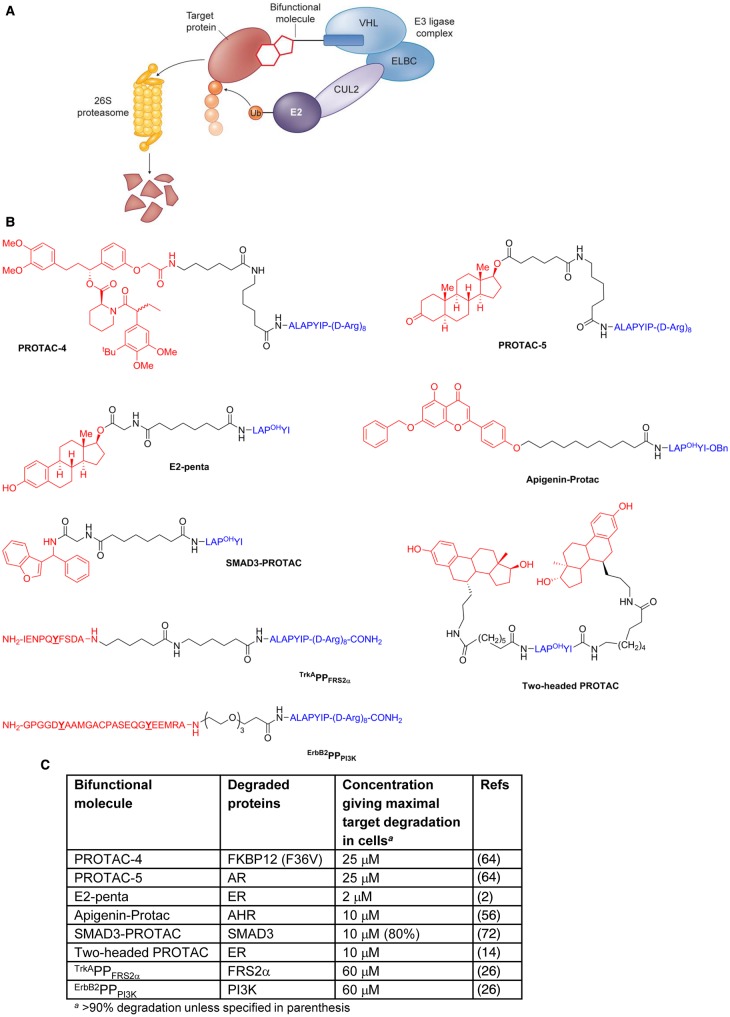
Selected bifunctional molecules hijacking the VHL E3 ligase using peptide motifs to target VHL. (**A**) Cartoon showing the complexes involved in VHL-dependent ubiquitination (ELBC, elongin B–elongin C heterodimer; CUL2, cullin2; E2, E2 ubiquitin ligase; Ub, ubiquitin). (**B**) Structures of bifunctional molecules showing the affinity groups targeting proteins for degradation (red), linker motifs (black), and peptide motifs targeting the VHL E3 ligase (blue). Y denotes sites of intracellular phosphorylation. P^OH^, hydroxyproline. (**C**) Proteins targeted for degradation by selected bifunctional molecules and the concentrations used in cellular assays where maximal target depletion was observed (AHR, aryl hydrocarbon receptor; AR, androgen receptor; ER, estrogen receptor; FKBP12, FK506-binding protein 12; FRS2α, fibroblast growth factor receptor substrate 2; PI3K, phosphatidylinositol-4,5-bisphosphate 3-kinase; SMAD3, SMAD family member 3).

An alternative approach to achieve targeted protein degradation with chemical compounds involves the use of larger bifunctional molecules consisting of distinct substrate-binding and E3 ligase-binding groups conjugated by a linker; as first demonstrated for the recruitment of the Skp1–CUL–F-box complex containing Hrt1 (SCF) ubiquitin ligase to degrade methionine aminopeptidase-2 (MetAP-2) [61]. The conjugate molecule serves to assemble a ternary complex between the E3 ligase, target protein, and probe molecule, allowing the E3 ligase complex to ubiquitinate the non-natural substrate and promote proteasome-dependent degradation [illustrated for the von Hippel–Lindau (VHL) E3 ligase in Figure 4A]. Whereas binding of the low molecular mass IMiD molecules discussed above results in a subtle variation in the E3 ligase receptor-binding surface, changing the affinity and specificity for protein–protein interactions with substrates, the bifunctional molecule approach creates a spatially distinct small-molecule binding site for protein substrates proximal to, but separate from, the E3 ligase itself. For a productive ternary species to be formed, the bifunctional molecule must contain a selective ligand for the target protein of interest with a suitable position for attachment of a recognition group for the E3 ligase via a linker group, without substantial loss of target affinity. In addition, the proximity and orientation of the E3 ligase and target protein in the ternary complex must be permissive for target protein ubiquitination.

Recruitment of the VHL E3 ligase to induce degradation of targeted proteins is so far the most extensively explored approach to hijacking the ubiquitin–proteasome system, following the first demonstration of proof-of-concept by PROteolysis TArgeting Chimeras (PROTACs) from Deshaies, Crews and colleagues ([61]; reviewed in refs [57,9,71,36]). Initially, small peptide chains were used to mimic the protein–protein interaction between the VHL E3 ubiquitin ligase complex and the endogenous substrate, hypoxia-inducible factor 1α (HIF1α). The minimal recognition domain amino acid sequence ALAPYIP [27] contains a key central proline motif, analogous to P564 in HIF1α, that becomes hydroxylated in cells ultimately leading to ubiquitination and degradation of HIF1α under normoxic conditions [18].

The first cell-permeable bifunctional molecules included PROTAC-4 (Figure 4B), which was designed to target the F36V mutation of FK506-binding protein (FKBP12) [64]. The VHL-interacting seven amino acid peptide sequence was appended to the ligand AP21998, which targets a mutant FKBP12. To confer cell permeability and a degree of stability to proteolysis, a poly-d-arginine tag was also introduced to PROTAC-4. Consequently, F36V mutant FKBP12 protein fused to a green fluorescent protein (GFP) tag expressed in HeLa cells was shown in a fluorescence assay to be degraded by treatment with PROTAC-4 at a concentration of 25 µM over 2.5 h. As further proof that the bifunctional molecules could be effective at inducing selective protein degradation in cells, PROTAC-5 (Figure 4B) was designed using the same seven amino acid and polyarginine peptide. In this instance, the peptide motif was conjugated to dihydrotestosterone to target the androgen receptor (AR). Fluorescence analysis of GFP-labelled AR in HEK293 cells showed protein degradation after 1 h exposure to PROTAC-5 at concentrations of 25 µM and above. Additionally, pretreatment with the proteasome inhibitor epoxomicin prevented degradation of the GFP–AR protein, as did treatment alone with either testosterone or the truncated peptide–polyarginine construct.

The bifunctional molecule E2-penta (Figure 4B) was used to hijack the VHL VBC–CUL2 complex to ubiquitinate and degrade estrogen receptor α (ERα) in a potential approach to antiangiogenesis [2]. Endogenous 17β oestradiol (E2) promotes angiogenesis via ERα by direct endothelial cell proliferation, migration, and up-regulation of basic fibroblast growth factor and vascular endothelial growth factor (VEGF) and their receptors. Introduction of the pre-hydroxylated proline motif, the key recognition factor in the degradation of HIF1α, allowed the chain to be shortened to a pentapeptide that showed improved potency over longer peptide analogues, presumably due to better cell permeability. Inhibition of VEGF-induced sprouting of human umbilical vein endothelial cells occurred following incubation with 2 µM concentrations of E2-penta over 24 h. This was the first report of using PROTACs to perturb downstream biological function and provided proof of concept for using such chemical probes to degrade target proteins and study the biological consequences.

The aryl hydrocarbon receptor (AHR) is present in the cytoplasm as part of a chaperone complex and has been implicated in tumour promotion and progression. Apigenin-Protac (Figure 4B), containing the hydroxylated pentapeptide VHL-recognition domain, was found to bind significantly less strongly to AHR than the unconjugated AHR ligand apigenin (Apigenin-Protac IC_50_ = 4 µM, compared with Apigenin IC_50_ = 0.3 µM) [56]. Despite this 10-fold decrease in binding affinity, Apigenin-Protac was found to degrade AHR protein levels in neonatal primary human keratinocytes at concentrations of 10 µM after 12 h incubation, in addition to inhibiting expression of 2,3,7,8-tetrachlorodibenzo-*p*-dioxin-induced cytochrome P450 1A1 (CYP1A1) protein, a biomarker of AHR function. Usefully, a negative control bifunctional molecule was also reported, whereby the hydroxyproline amino acid critical for VHL recognition was replaced with alanine, and was shown to have no effect on AHR degradation.

In a recent example, the hydroxylated pentapeptide VHL-recognition motif was used to target SMAD family member 3 (SMAD3), a key signalling protein in renal fibrosis, for degradation [72]. In the absence of a suitable small-molecule SMAD3 inhibitor, an *in silico* docking screen of a commercial library was performed. Of the hits thought possibly suitable as PROTAC components and confirmed by surface plasmon resonance as SMAD3 ligands, a benzofuran ligand with moderate binding affinity (*K*_d_ = 45 µM) and slow off-rate was selected for inclusion in the bifunctional conjugate Smad3-Protac (Figure 4B). The molecule was found to decrease basal SMAD3 protein expression in both renal fibroblast (NRK-49F) and renal mesangial cells (HMC) at a 200 µg/ml concentration, representing an approximate 100-fold reduction in potency compared with the activity observed in human renal carcinoma ACHN cell lysates, and illustrating the difficulty of routinely obtaining high cell permeability with large bifunctional molecules containing a significant peptidic character.

A two-headed approach to the design of PROTAC-type conjugates was explored in the context of ER degradation [14]. The two-headed PROTAC (Figure 4B) was found to have a 3-fold improved binding affinity for ER in comparison with two control PROTAC molecules, each containing a single ER-targeting head group at either the N- or C-terminus of the VHL-targeting pentapeptide. The linker length between the ER ligand and the VHL-targeting peptide in the PROTAC was found to be important for activity [15]. Although observed to have poorer solubility than the monomeric counterparts, two-headed PROTAC had improved ability to induce ER degradation at 10 µM over 48 h incubation, with a greater than 5-fold enhancement of potency over monomeric counterparts. Importantly, to demonstrate the mechanism of action, the control compound where the central hydroxyproline VHL-recognition amino acid was replaced with norleucine did not cause ER degradation under the same conditions, nor did degradation occur with two-headed PROTAC in the presence of the proteasome-specific inhibitor epoxomicin.

An exciting extension of the bifunctional conjugate approach whereby the conditional degradation of a protein target occurs only subsequent to an intracellular phosphorylation event has been developed [26]. As an example, the tyrosine residue highlighted in ^TrkA^PP_FRS2α_ (Figure 4B) must first be phosphorylated by the kinase, tropomyosin receptor kinase A (TrkA), before degradation of the target, fibroblast growth factor receptor substrate 2 (FRS2α), is observed. In the presence of nerve growth factor (NGF), TrkA undergoes NGF-induced dimerisation and *trans*-autophosphorylation. The fully active phosphorylated tyrosine kinase can, in turn, bind and phosphorylate many substrates, including FRS2α. ^TrkA^PP_FRS2α_ was designed to incorporate a 10 amino acid recognition sequence around the tyrosine residue phosphorylated in TrkA, a seven amino acid recognition sequence to bind to VHL, and a polyarginine sequence to improve cell permeability. In the presence of ^TrkA^PP_FRS2α_, NGF-treated PC12 cells incorporated radiolabelled ^32^P phosphate into the ^TrkA^PP_FRS2α_ conjugate, a phenomenon not observed in the absence of NGF. Incubating PC12 cells in the presence of NGF for 7 h showed an increase in phosphorylation of FRS2α with no loss of protein levels. However, including 40 µM ^TrkA^PP_FRS2α_ gave a 50% reduction in FRS2α protein levels and loss of downstream extracellular signal-regulated kinase (ERK)1/2 phosphorylation, without any associated decrease in overall ERK1/2 protein levels. Pretreatment of PC12 cells with epoxomicin was used to confirm dependence on the ubiquitin-proteasome pathway and indeed caused accumulation of FRS2α and higher molecular mass FRS2α–ubiquitin conjugates in the presence of NGF and ^TrkA^PP_FRS2α_. The requirement for TrkA phosphorylation of ^TrkA^PP_FRS2α_ to induce degradation was supported by use of a Phe/Tyr residue swap in a control bifunctional molecule, thus removing the site of potential phosphorylation, and giving no FRS2α degradation or reduction in ERK1/2 phosphorylation in the presence of NGF.

A further example of the conditional degradation strategy was described using ^ErbB2^PP_PI3K_ (Figure 4B) to deplete phosphatidylinositol-3-kinase (PI3K). ErbB3 phosphorylation in a heterodimer complex between ErbB2 and ErbB3 epidermal growth factor receptor tyrosine kinases at the cell membrane results in recruitment of PI3K through binding to phospho-ErbB3. The design of the phosphoPROTAC ^ErbB2^PP_PI3K_ therefore contained a 24 amino acid sequence taken from the PI3K-binding segment of ErbB3. Both the highlighted tyrosine residues in Figure 4B are phosphorylated by ErbB2, leading to recruitment of PI3K for degradation. Accordingly, ^ErbB2^PP_PI3K_ induced depletion of PI3K expression in MCF-7 cells at concentrations of 40 µM and above. Replacing the Tyr residues in ^ErbB2^PP_PI3K_ with Phe as before confirmed the requirement for tyrosine phosphorylation to occur before observing any reduction in PI3K protein levels. Additionally, a mouse study using 10 mg/kg i.p. dosing of ^ErbB2^PP_PI3K_ in OVCAR8 subcutaneous xenograft tumours demonstrated a 40% reduction in tumour weight relative to control.

**Figure 5.**
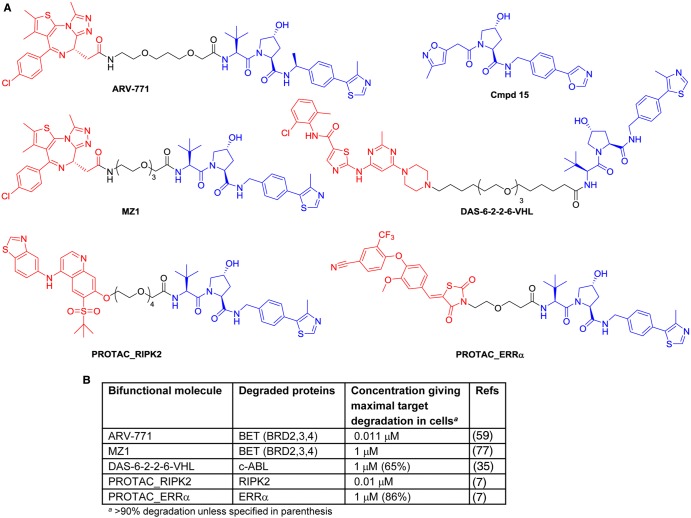
Selected bifunctional molecules hijacking the VHL E3 ligase using small-molecule VHL inhibitors. (**A**) Structures of bifunctional molecules hijacking the VHL E3 ligase, showing the affinity groups targeting proteins for degradation (red), linker motifs (black), and VHL E3 ligase-targeting motif (blue). (**B**) Proteins targeted for degradation by selected bifunctional molecules and the concentrations used in cellular assays where maximal target depletion was observed (BET, bromodomain and extra-terminal family of proteins; BRD4, bromodomain-containing protein 4, member of the BET family; c-ABL, Abelson tyrosine kinase; RIPK, receptor-interacting serine/threonine-protein kinase 1; ERRα, estrogen-related receptor α).

While cellular and *in vivo* efficacy has been shown in peptide-derived VHL affinity groups as discussed above, the use of a non-peptide, selective small-molecule VHL-recognition motif could offer many advantages in regard to improved potency, metabolic stability, and permeability. The rational design of small-molecule VHL ligands such as cmpd 15 (Figure 5A) and determination of its crystal structure bound to VHL [8] has led to the development of submicromolar VHL ligands with reduced peptide character [20]. Incorporation of a *t*-butyl group in the VHL-binding component, as in Protac_ER-related receptor-α (ERRα; Figure 5A), has been found to give high affinity for VHL [7]. Protac_ERRα gave 50% degradation (DC_50_) of ERRα in MCF-7 cells at 100 nM, whereas the epimeric proline alcohol, used as a negative control as it no longer binds to VHL, gave ∼20% degradation at the same concentration. In addition, Protac_ERRα showed depletion of ERRα *in vivo* when dosed at 100 mg/kg i.p., three times per day, to mice bearing MDA-MB-231 xenograft tumours. Similarly, Protac_RIPK2 (Figure 5A) was shown to be particularly potent in inducing degradation of RIPK2, with a DC_50_ of 1.4 nM in human THP-1 monocytes and complete depletion at 10 nM. Of note was a biphasic response at high concentrations of Protac_RIPK2, where protein levels of RIPK2 recovered to basal levels, an effect attributed to an inability to form the proposed ternary complex in the presence of excess Protac_RIPK2 concentrations, which increases binary complex formation and thus stops degradation. The epimeric proline alcohol of Protac_RIPK2 made as a negative control did not show any degradation at concentrations up to 10 µM. Both active Protac_RIPK2 and the inactive epimeric alcohol were used to provide evidence to support the formation of a ternary complex through chemoproteomic pull-down and immunoprecipitation experiments. Moreover, Protac_RIPK2 was found to act catalytically *in vitro*, inducing super-stoichiometric ubiquitination of RIPK2. For both Protac_ERRα and Protac_RIPK2, a dependence on proteasomal degradation in cells was confirmed by pretreatment with the proteasome inhibitor epoxomicin, resulting in blockade of the degradation of ERRα and RIPK2. The specificities of Protac_ERRα and Protac_RIPK2 towards degradation of their intended target proteins were assessed by cellular expression proteomics in cancer cells, monitoring ∼7600 proteins. For Protac_ERRα, only degradation of ERRα and (after prolonged exposure) breakpoint cluster region (BCR) protein were observed, while Protac_RIPK2 showed a similar specificity with RIPK2 and the kinase MAPKAPK3 as the only proteins degraded.

The treatment of chronic myelogenous leukaemia (CML) by inhibition of the oncogenic protein kinase BCR-ABL using small-molecule drugs such as imatinib, dasatinib, or bosutinib has been highly effective. There is, however, a need for lifelong treatment with the drug, speculated to be due to the ability of BCR-ABL to act as a scaffolding protein in a compensatory pathway that produces leukaemic stem cells in spite of kinase inhibition. Degradation of BCR-ABL therefore presents an alternative approach to modulation of this important target [35]. Of the VHL-binding conjugates synthesised with varying linker group lengths, DAS-6-2-2-6-VHL (Figure 5A) showed degradation of the wild-type protein Abelson tyrosine kinase (c-ABL) at 1 µM in K562 human CML cells over 24 h. However, no associated degradation of the oncogenic BCR-ABL fusion protein was observed, despite potent binding of dasatinib to both proteins, and in contrast with the effects of related conjugates designed to recruit the CRBN E3 ligase complex, discussed in detail in ‘Bifunctional molecules hijacking the MDM2, cIAP, and CRBN E3 ligases’. Thus, a clear need to optimise not only the target-binding motif and linker length in the bifunctional molecules, but also the choice of E3 ligase for recruitment in a given degradation target context, has been demonstrated.

The bifunctional molecule ARV-771 (Figure 5A) has been found to induce potent degradation of the bromodomain and extra-terminal domain (BET) family of proteins, potentially of great interest in the treatment of prostate cancer, and castration-resistant prostate cancer in particular [59]. ARV-771 was constructed by conjugating JQ-1, a small-molecule BET inhibitor, to a hydroxyproline-based VHL-binding domain via an optimised linker group. Degradation of the BET family of proteins, bromodomains (BRD)2, 3, and 4, by ARV-771 was observed in 22Rv1, VCaP, and LnCaP95 human prostate cancer cell lines at concentrations of 5–10 nM. Loss of expression of c-MYC, a downstream effector of BET, was also observed at both the protein and mRNA levels at <10 nM concentrations for ARV-771. An inactive control diastereoisomer ARV-766, containing the epimeric proline alcohol unable to bind VHL, was found to have comparable binding affinity for the BET proteins to that seen for ARV-771 and JQ-1. ARV-766 showed a negligible effect on c-MYC mRNA levels at 1 µM despite being a potent BET inhibitor in its own right, suggesting poor permeability. Given that the active conjugate ARV-771 may be expected to have comparable cell permeability, the low nanomolar cellular activity observed for ARV-771 has been hypothesised to be due to the potential catalytic action of the bifunctional species. Additionally, ARV-771 was shown to induce BRD4 and c-MYC degradation in 22Rv1 tumour xenografts in mice, and more importantly led to tumour regression in the 22Rv1 model following daily subcutaneous dosing of 30 mg/kg.

Variation in the linker length between JQ-1 and the VHL-binding motif has led to the discovery of linker-dependent selectivity within the BET family of proteins [77]. MZ1 (Figure 5A) and JQ-1 were shown to have comparable binding affinities for the BET family of proteins; however, MZ1 was found to preferentially degrade BRD4 over BRD2 and BRD3 at a concentration of 1 µM in HeLa cells over 24 h. In the U2OS cell line, MZ1 demonstrated dose- and time-dependent selective degradation of BRD4 over BRD2 and BRD3. Furthermore, MZ1 was shown to have no effect on endogenous levels of VHL or HIF1α, an important factor in the potential use of such bifunctional molecules as drug therapies.

### Bifunctional molecules hijacking the MDM2, cIAP, and CRBN E3 ligases

**Figure 6.**
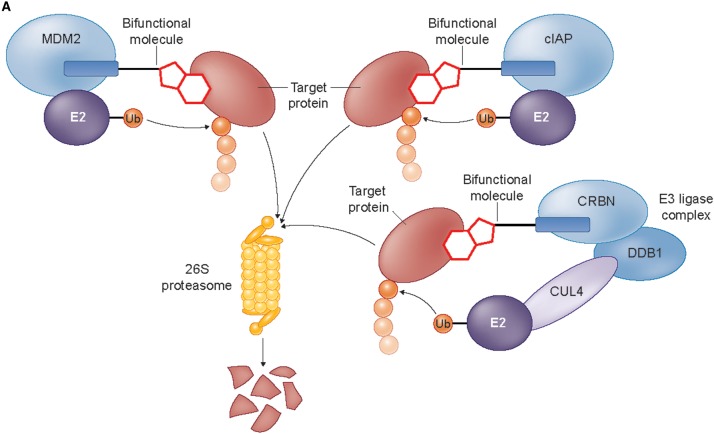

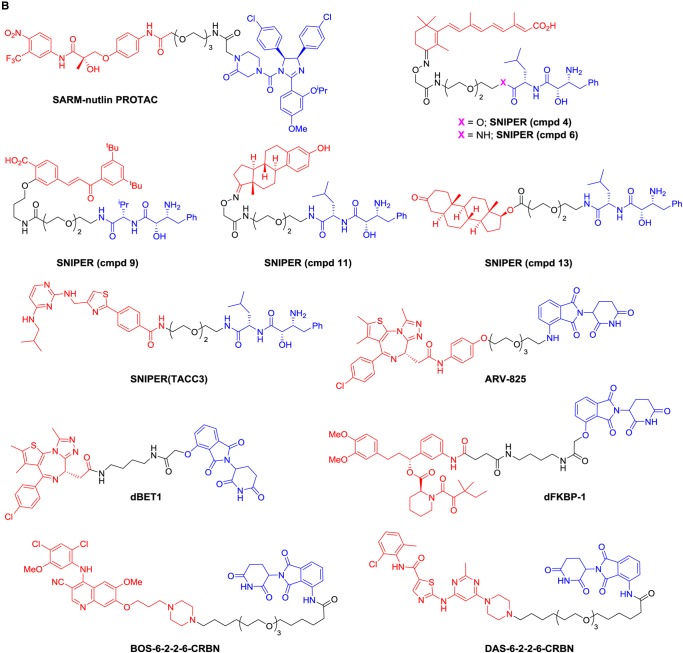

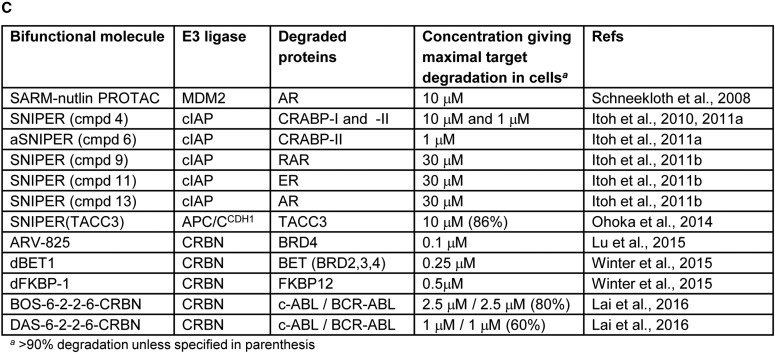
Selected bifunctional molecules hijacking the MDM2, cIAP1, and CRBN E3 ligases. (**A**) Cartoon showing the complexes involved in MDM2-, cIAP-, and CRBN-dependent ubiquitination (cIAP, cellular inhibitor of apoptosis protein; CRBN, cereblon; CUL4, cullin 4; DDB1, DNA damage-binding protein 1; E2, E2 ubiquitin ligase; MDM2, mouse double minute 2 homologue; Ub, ubiquitin). (**B**) Structures of bifunctional molecules showing the affinity groups targeting proteins for degradation (red), linker motifs (black), and small-molecule motifs that recruit the E3 ligases (blue). (**C**) Proteins targeted for degradation by selected bifunctional molecules, the E3 ligases recruited, and the concentrations used in cellular assays where maximal target depletion was observed (APC/C^CDH1^, anaphase-promoting complex/cyclosome in complex with CDH1; AR, androgen receptor; BCR-ABL, breakpoint cluster region — Abelson kinase fusion; BRD4, bromodomain 4; c-ABL, Abelson murine leukaemia viral oncogene cellular homologue; CRBP, cellular retinoic acid-binding protein; ER, estrogen receptor; FKBP12, FK506-binding protein 12; RAR, retinoic acid receptor; TACC3, transforming acidic coiled-coil-3).

E3 ligases other than VHL have been successfully hijacked using bifunctional molecules. The first non-peptidic E3 ligase-targeting group was used to direct MDM2, whose natural substrates include the tumour-suppressor TP53, to degrade the AR (Figure 6A) [65]. A selective androgen receptor modulator (SARM) with nanomolar affinity for AR was conjugated to the MDM2–TP53 interaction inhibitor nutlin to generate the SARM-nutlin PROTAC, which decreased AR expression in HeLa cells in a proteasome-dependent manner at a 10 µM concentration (Figure 6B,C). However, interpretation of the cellular activity of the SARM-nutlin PROTAC is complicated as AR is known to be a direct substrate of MDM2 [40], and nutlin itself induces ubiquitination and degradation of AR in cancer cells [41], raising the possibility of a direct modulatory effect of the SARM-nutlin bifunctional molecule on AR degradation independent of ternary complex formation [31,70].

While the cellular potency for SARM-nutlin PROTAC was similar to that seen for VHL-targeting peptidic PROTACs, subsequent iterations using small-molecule E3 ligase-binding groups have achieved breakthroughs in cell activity to the nanomolar range, notably for non-peptidic molecules hijacking VHL (Figure 5A,B) and CRBN (Figure 6B,C). A suite of bifunctional molecules, termed specific and non-genetic IAP-dependent protein erasers (SNIPERs), was developed by Hashimoto and colleagues to redirect the activity of the E3 ligase cIAP1 that degrades caspase proteins and is overexpressed in many cancer cells (Figure 6A). The conjugates make use of derivatives of bestatin methyl ester, a cell permeable small molecule that binds to cIAP1 to promote autoubiquitination and degradation [67]. Proof-of-concept was achieved with SNIPER cmpd 4 (Figure 6B) that linked a bestatin ester moiety to all-*trans* retinoic acid to capture the cellular retinoic acid-binding proteins (CRABP-I and -II) [29]. Proteasome-dependent degradation of CRABP-I/II was observed in the range of 1–10 µM in cells. Interestingly, varying the linker length in SNIPER cmpd 4 changed the relative efficiency of degradation of CRABP-I and -II, with CRABP-I better depleted by a conjugate with longer linker. Reduced expression of CRABP-II in IMR-32 human neuroblastoma cells by SNIPER cmpd 4 gave a dose-dependent reduction in cell motility.

To avoid the concomitant autoubiquitination and degradation of cIAP promoted by bestatin ester derivatives, the amide-linked SNIPER cmpd 6 was developed to retain cIAP binding but abolish autodegradation [30]. SNIPER cmpd 6 promoted depletion of CRABP-II from 1 µM in cells with no effect on cIAP levels or apparent inhibition of cIAP endogenous function. A comparison of the effects of SNIPER cmpd 4 and cmpd 6 in IMR-32 neuroblastoma cells showed that combined CRABP-II and cIAP degradation was advantageous for inhibition of cell growth and induction of apoptosis. Amide-linked SNIPERs have also been described for targeted depletion of nuclear receptors, namely retinoic acid receptor (cmpd 9, Figure 6B), ER (cmpd 11, Figure 6B), and AR (cmpd 13, Figure 6B), albeit with reduced cell potency (Figure 6C) [31].

A bifunctional molecule SNIPER(TACC3) was designed to induce degradation of the mitotic spindle regulatory protein transforming acidic coiled-coil-3 (TACC3) (Figure 6B) [54]. While ubiquitination- and proteasome-dependent depletion of TACC3 was observed in cells treated with SNIPER(TACC3), this was unexpectedly found not to be due to recruitment of the cIAP E3 ligase as anticipated. Instead, mechanistic studies showed that the degradation was mediated by the ubiquitin ligase, anaphase-promoting complex/cyclosome in complex with CDH1 (APC/C^CDH1^). APC/C^CDH1^ is the E3 ligase responsible for TACC3 degradation in unperturbed cells, but a physical interaction between the SNIPER(TACC3) molecule and APC/C^CDH1^ was demonstrated by a thermal stability assay, suggesting that both SNIPER(TACC3)-dependent and independent mechanisms may contribute to TACC3 degradation. The cause of this switch in E3 ligase recruitment by the bestatin-linked bifunctional molecule remains unclear. These findings indicate that a consistent mechanism of action is not guaranteed for newly designed bifunctional molecules, and that proof of the mechanism is required to accompany their use as chemical probes.

High cellular potency for targeted protein degradation has been achieved with bifunctional molecules that recruit CUL4^CRBN^ (Figure 6A). ARV-825 PROTAC (Figure 6B), consisting of a high-affinity triazolo-diazepine related to the potent BRD4 inhibitor JQ-1 conjugated to a pomalidomide derivative, was designed to promote CRBN-dependent degradation of BRD4, a member of the BET family of epigenetic reader proteins [46]. The conjugate retained high affinity for BRD4 (*K*_d_ = 28 nM for bromodomain 1 of BRD4) and showed complete degradation of BRD4 in cells at a 100 nM concentration (Figure 6C), suggesting that the bifunctional molecule is acting catalytically with respect to recruiting BRD4. Of note, the affinity of pomalidomide for CRBN is only ∼3 µM, again indicating that each bifunctional molecule participates in more than one recruitment cycle, and that transient linking of the target protein and E3 ligase is sufficient for efficient ubiquitination. A bell-shaped concentration response curve was observed for degradation of BRD4 in cells, consistent with the formation of a trimeric BRD4/ARV-825 PROTAC/CRBN complex as the active species promoting ubiquitination. The observed sustained protein degradation was in contrast with the effects of unconjugated BRD4 ligands that lead to hyperaccumulation of BRD4 on prolonged exposure. As a result, increased downstream effects on suppression of the c-MYC protein, inhibition of B-cell proliferation, and induction of apoptosis were seen with the conjugate compared to the effects of the unconjugated BRD4 ligands.

In a parallel approach, the conjugation of the BRD4 inhibitor JQ-1 to a pomalidomide/thalidomide hybrid gave the bifunctional molecule dBET1 (Figure 6B) that retained the selectivity of JQ-1 for BRD4 binding within the BET family [74]. Taking advantage of the crystal structures available for both BRD4 and CRBN with ligands bound, the linker length for dBET1 was designed based on *in silico* modelling of the ternary complex. Extensive depletion (>85%) of BRD4 by dBET1 was seen at 100–250 nM in human cells for up to 18 h (Figure 6C). Some recovery of protein levels was seen on longer exposure, suggestive of chemical instability of the bifunctional molecule. Proteomic assessment of the effects of dBET1 and the unconjugated BRD4 inhibitor JQ-1 showed highly similar, selective effects of both molecules. Out of 7429 proteins monitored, the bifunctional molecule dBET1 elicited depletion of MYC and PIM1 as expected based on the downstream effects of depletion of BRD4 and only three other proteins (BRD2, BRD3, and BRD4), consistent with the specificity of the JQ-1 ligand for the BET family. However, enhanced apoptotic effects were seen for dBET1 in cancer cell lines and primary human acute myeloid leukaemia cells. The effects of targeted degradation of BRD4 were investigated *in vivo* following intraperitoneal dosing of dBET1 to mice bearing xenograft human MV4-11 leukaemia cells. Inhibition of tumour growth relative to untreated controls was observed, with pharmacodynamic evidence of BRD4 depletion in treated tumours seen. A head-to-head comparison of equimolar amounts of dBET1 and JQ-1 dosed in a model of disseminated leukaemia showed a ∼3-fold increase in antitumour activity for the bifunctional molecule over the simple BRD4 ligand.

Conjugation to CRBN-recruiting groups has also been demonstrated for the FKBP12 ligand steel factor (SLF), giving the potent promoter of FKBP12 degradation, dFKBP-1 (Figure 6B) [74]. CRBN-recruiting groups were also linked to the BCR-ABL receptor tyrosine kinase inhibitors bosutinib and dasatinib, to give BOS-6-2-2-6-CRBN and DAS-6-2-2-6-CRBN, respectively (Figure 6B) [35]. In the latter study, Crews and colleagues adopted a modular approach to vary the affinity group (kinase inhibitor), linker structure, and E3 ligase recruiting group of the PROTAC molecules. This allowed a direct comparison between recruitment of VHL and CRBN E3 ligases for the same targets. The crystal structures of ligand-bound c-ABL were used to select the attachment points for the linkers, and the derived bifunctional molecules maintained nanomolar affinity for BCR-ABL despite a general fall in potency. Intriguingly, none of the conjugates made that targeted VHL led to BCR-ABL degradation in cells (see ‘Hijacking the von Hippel-Lindau E3 ligase’). This was not due to lack of BCR-ABL binding and was speculatively attributed to an unproductive orientation of the VHL E3 ligase in the trimeric complexes. In contrast, the conjugates recruiting CRBN (e.g. BOS-6-2-2-6-CRBN and DAS-6-2-2-6-CRBN) elicited potent degradation of BCR-ABL in cells, showing that the oncogenic tyrosine kinase has varying levels of susceptibility to modification by different hijacked E3 ligases. As the efficiency and selectivity of target degradation was also found to depend on the kinase inhibitor moiety, the authors suggest that a modular approach to an array of bifunctional molecule designs, to optimise empirically the best combination of target and E3 ligase-binding functionalities, may be advantageous when seeking new probes for targeted protein degradation. Such studies require rapid syntheses of the components of the bifunctional molecules, such as that recently demonstrated for amine-substituted phthalimide derivatives [42].

A recent publication shows how high molecular mass bifunctional molecules can be self-assembled *in situ* in cells from smaller components using bio-orthogonal ‘click’ chemistry to link separate precursors containing the CRBN-binding and protein-targeting functionalities [37]. These click-formed proteolysis-targeting chimeras (CLIPTACs) recruiting CRBN were prepared by conjugation of thalidomide to JQ-1 (JQ1-CLIPTAC) or to a covalent ERK1/2 inhibitor (ERK1/2-CLIPTAC) to achieve depletion of BRD4 or ERK 1 and 2, respectively. A potential advantage for achieving cell penetration with smaller components was demonstrated by the observation that preformed CLIPTACs gave no target degradation, while combination of the smaller click-enabled thalidomide moiety (10 µM) with click-enabled JQ-1 (3 µM) gave complete BRD4 depletion after 18 h.

### Targeted protein degradation through recruitment of HSP70 molecular chaperones or direct binding to the 20S proteasome

**Figure 7.**
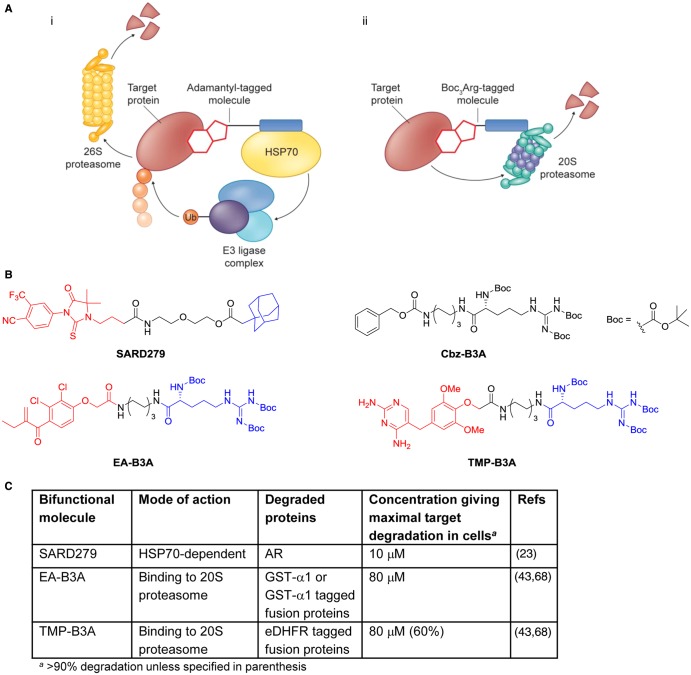
Selected bifunctional molecules directing target degradation through binding of HSP70 or the 20S proteasome. (**A**) Cartoons showing the complexes involved in (i) HSP70-dependent protein degradation mediated by a hydrophobic adamantyl tag (HSP70, heat shock protein 70; Ub, ubiquitin) and (ii) direct recruitment of the 20S proteasome by Boc_3_Arg tags. (**B**) Structures of bifunctional molecules that direct target protein degradation through binding of HSP70 (SARD279) or the 20S proteasome (EA-B3A, TMP-B3A) showing the affinity groups targeting proteins for degradation (red), linker motifs (black), and small-molecule motifs targeting degradation machinery (blue). (**C**) Mode of action and proteins targeted for degradation, and the concentrations used in cellular assays where maximal target depletion was observed (AR, androgen receptor; eDHFR, *E. coli* dihydrofolate reductase; GST-α1, glutathione *S*-transferase).

The bifunctional molecules described above affect protein degradation by direct binding to E3 ligase complexes. Protein ubiquitination and degradation can also be achieved through hijacking the unfolded protein response using very lipophilic small-molecule tags to recruit molecular chaperones, such as HSP70 family members that recognise the exposed hydrophobic cores of unfolded proteins (Figure 7Ai). HSP70 and co-chaperone binding direct the tagged protein for E3 ligase-mediated ubiquitination and degradation as though it was an unfolded client. First demonstrated for Halotag proteins [52] using an adamantyl hydrophobic tag, this approach was extended to bifunctional adamantyl derivatives including the selective androgen receptor degrader SARD279 (Figure 7B) [23]. Conjugation of the high-affinity AR agonist RU59063 to the adamantyl group reduced the binding affinity for AR by 37-fold, but led to degradation of AR in LNCaP human prostate cancer cells at low micromolar concentrations, while no AR degradation was seen upon treatment with unconjugated RU59063. Selective degradation of AR over the glucocorticoid receptor (GR) was induced by SARD279, consistent with the selectivity of the RU59063 affinity group for AR over GR. Increasing the cellular expression of HSP70 isoforms using the HSP90 inhibitor geldanamycin enhanced the SARD279-dependent AR degradation, suggesting a role for HSP70 in mediating degradation of the AR–SARD279 complex. SARD279 showed more potent inhibition of AR-dependent gene expression (IC_50_ = 156 nM) than that for AR degradation, indicating a dual mode of activity through competitive inhibition of AR transactivation and AR depletion. Importantly, in contrast with competitive AR antagonists, eliminating AR protein with SARD279 was found to be antiproliferative in AR-dependent prostate cancer cell lines and also in castration-resistant prostate cell lines, whether resistance to antiandrogens resulted from increased androgen levels or from the F876L AR mutation that converts antagonists into agonists [23].

A distinct approach to targeted protein degradation was achieved using ligands linked to arginine triply-protected with the bulky, lipophilic *tert-*butyloxycarbonyl (Boc) group [43]. Thus, the Boc_3_-protected arginine (B3A) conjugate of the covalent glutathione *S*-transferase (GST) inhibitor ethacrynic acid (EA-B3A, Figure 7B) induced degradation of GST-fusion proteins in lysates from HeLa cancer cells and of endogenous GST-π as well as ectopically expressed GST-fusion proteins in Cos-1 or HeLa cells. Covalent attachment of the affinity group to the target protein appeared to enhance the degradation potency of the bifunctional molecules. The conjugate TMP-B3A (Figure 7B) based on trimethoprim, a reversible, non-covalent inhibitor of bacterial dihydrofolate reductase (eDHFR), was tested head-to-head with EA-B3A for the ability to promote degradation of an ectopically expressed eDHFR–HA–GST-α1 fusion protein in HeLa cells. While 80 µM EA-B3A gave complete depletion of the fusion protein in under 2 h, only 25% removal of protein was achieved by 80 µM TMP-B3A after 5 h.

In contrast with other targeted degradation approaches, the mechanism of B3A-promoted degradation was found not to require ubiquitination of the target protein, nor the involvement of the 26S proteasome [43,68]. Neither did binding of B3A conjugates intrinsically destabilise the target proteins and induce unfolding. Instead, a direct non-covalent interaction of the B3A group with the 20S proteasome was uncovered, and purified 20S proteasome was found to be sufficient for target protein degradation in cell-free systems. Thus, this represents the first example of using a bifunctional small molecule for direct targeting of a protein to the 20S proteasome for degradation (Figure 7Aii) and is an exciting addition to the growing repertoire of pharmacological techniques to control protein degradation.

The two approaches outlined above offer complimentary alternatives to the hijacking of specific E3 ligases to achieve targeted protein degradation. The hydrophobic and B3A tags do not have the same potential to alter endogenous E3 ligase substrate specificity as may be seen with VHL- or CRBN-directing tags, but there are other potentially interfering biological outputs from the functional groups in the conjugate molecules that may complicate interpretation of cellular experiments. HaloTag protein modification with adamantyl-derived groups can induce a transient unfolded protein response [58]. While it is not demonstrated that this applies for the reversible, bifunctional molecules, the activation of HSP70 isoforms could complicate the phenotype seen on targeted degradation. On the other hand, the simple B3A-containing molecule Cbz-B3A (Figure 7B), which lacks a specific affinity group, has been shown to block eIF4E-binding protein 1-dependent translation through an as-yet-uncharacterised interaction with ubiquilins [12]. These inevitable caveats of reagent selectivity notwithstanding, pharmacological degradation of putative targets by more than one of the complementary approaches available would help to rule out off-target effects.

### Experimental approaches to characterising bifunctional modulators of E3 ligase activity

The discovery and validation of new bifunctional molecules to hijack E3 ligases requires characterisation of their mode of action, especially if they are to be used effectively as tools to explore the biological consequences of specific protein depletion. This mirrors the validation of classical small-molecule chemical probes [75]. The common experimental approaches applied to characterise PROTACs, SNIPERs and other bifunctional molecules are summarised in Table 1. These typically provide evidence for engagement of the target protein(s) and E3 ligase, trimeric complex formation, ubiquitin- and proteasome-dependent degradation of the target, specificity for the target, and differentiation of the bifunctional compound from the component binding groups. In most cases, these experiments are supported by the parallel discovery and profiling of negative control compounds, typically bifunctional molecules where one of the binding groups has been rendered ineffective, as well as the use of competition experiments between the bifunctional probe and the small-molecule component binding groups. Details of these approaches are presented in many of the publications surveyed in ‘Hijacking E3 ligases for specific target degradation using bifunctional molecules’.

**Table 1:** Common experimental approaches to characterising bifunctional modulators of E3 ligase activity for their suitability as chemical tools

Characterisation	Experimental approaches
Evidence of target degradation	Cell-based assessment of target protein expression Dose-dependent depletion of target protein and quantification of potency (DC_50_, DC_90_, or similar)
Evidence of binding to target protein	Biochemical (cell-free) assay of binding/inhibition by the bifunctional molecule Cell-based assay for inhibition of function of the target protein by the bifunctional molecule Reduction in target degradation in cells by competition with the unconjugated affinity group and/or an alternative small molecule targeting the same binding site
Evidence for binding and recruitment of an E3 ligase	Reduction in target degradation in cells by competition with the unconjugated recruitment motif for the E3 ligase (e.g. pomalidomide for CRBN) Target degradation abolished in ligase-deficient cell lines
Evidence for ubiquitin-dependent degradation	Assay for ubiquitination of the target protein following immunoprecipitation from cells treated with the bifunctional molecule and a proteasome inhibitor (e.g. MG132)
Evidence for 26S proteasome-dependent degradation	Inhibition of probe-induced target degradation in the presence of a proteasome inhibitor (e.g. MG132, carfilzomib, and epoxomicin)
Evidence of a trimeric complex formation (E3 ligase — bifunctional molecule — target protein) mediating the observed effects	Bell-shaped concentration–response for target protein degradation in cells (may only be seen for potent probes) or in a cell-free proximity assay (e.g. AlphaScreen) Confirmation that target degradation induced by the bifunctional molecule is not induced by derivatives of the unconjugated affinity group or ligase recruitment group alone, or a mixture of the two. Recovery of the target protein following immunoprecipitation of the E3 ligase in cells treated with the bifunctional molecule Cell-free proximity assay using labelled ligase and target protein
Evidence of specificity for target degradation	Biochemical (cell-free) profiling of bifunctional molecule for binding/inhibition based on activities of the affinity group Cell-based assay for degradation of known/potential off-targets based on biochemical profiling of the bifunctional molecule or its unconjugated affinity group Cell-based assay for effects on the degradation of known substrates of the E3 ligase hijacked Differentiation of degradation promoted by the bifunctional molecule compared with negative control compounds where either the target affinity or ligase recruitment groups are replaced by structurally related, non-binding analogues (e.g. epimeric derivatives) Cellular expression proteomic profiling to determine effects on degradation.

## Conclusions and future perspectives

Two major recent breakthroughs have been achieved in the field of targeted protein degradation promoted by small molecules. One is the increased cell potency now routinely achievable using non-peptidic functionality in bifunctional molecules that engage E3 ligases to promote highly specific protein depletion [16]. This has enabled multiple proof-of-concept demonstrations of activity in animals with compounds promoting degradation of BET proteins or ERRα [74,7,59]. Importantly, two studies targeting BET protein degradation using bifunctional molecules that contain a BRD4-binding group showed antitumour activity in animal models, concomitant with the targeted protein degradation *in vivo* [74,59]. Optimising the physicochemical properties of high molecular mass bifunctional molecules to render them routinely suitable for human administration remains difficult [73], but these promising demonstrations of *in vivo* efficacy give impetus to solving this challenge.

The second breakthrough, which already addresses achieving drug-like physicochemical properties with small molecules that redirect E3 ligase activity, comes from understanding and exploiting the IMiD class of small-molecule modulators of CRBN substrate specificity. Here, the challenge is to learn how to predict and control the selectivity for neosubstrate degradation and to discern what limitations may exist to specificity. It is very encouraging that effective drug molecules are already in clinical use from this approach, and this indicates that the pleiotropic effects of multiple target protein degradation can be successfully used for therapeutic benefit.

The two chemical approaches to targeted protein degradation through modulation of the ubiquitin–proteasome pathway described in the present study are highly complementary, both in terms of their current use and future prospects. There are common considerations, for example the possible consequences of competition with endogenous substrates of the particular E3 ligase hijacked, and the need to carefully validate the mechanism of action of the chemical probes. With bifunctional molecules, the biological effect of proteasome-mediated depletion of the target protein needs to be differentiated from any direct effect of the affinity group on the target protein, especially when this is derived from a potent inhibitor or modulator in its own right. However, it is already clear that target depletion can have a more sustained activity than direct target inhibition and can overcome intrinsic feedback activation or overexpression of the target [46]. Exploiting the CRL4^CRBN^-mediated degradation of target proteins with small molecules that modulate the receptor surface will require understanding of the target degron sequence, biological context, and the development of chemical libraries that bind the tritryptophan cage in CRBN. Binding of substrates to ‘hotspot’ interaction sites on CRBN and the ability to affect degradation requires small molecules with low molecular mass and cell permeability that is a distinct advantage for developing drug-like molecules. On the other hand, there is limited further scope for further chemistry optimisation without perturbing the substrate specificity. For the bifunctional targeting approach, understanding the biology of the E3 ligases recruited, the optimal type of linker and how to assemble the bifunctional compounds is important, as well as the availability of specific binders for target proteins in the first place. Both complementary approaches therefore have advantages and disadvantages, but both will have an increasing role to play in the discovery of chemical probes for interrogating biological systems and the development of novel therapeutics through targeted protein degradation.

## Abbreviations

AHR: aryl hydrocarbon receptor
AR: androgen receptor
BCR: breakpoint cluster region
BRD: bromodomain
BET: bromodomain and extra-terminal domain
Boc: *tert-*butyloxycarbonyl
c-ABL: Abelson tyrosine kinase
CK1α: casein kinase 1α
CLIPTACs: click-formed proteolysis-targeting chimeras
CML: chronic myelogenous leukaemia
CRABP: cellular retinoic acid-binding protein
CRBN: cereblon
CRLs: cullin-RING ligases
CUL: cullin
CUL4: cullin 4
DCAFs: DDB1- and CUL4-associated factors
DDB1: DNA damage-binding protein 1
DLBCL: diffuse large B-cell lymphoma
DUBs: deubiquitinase enzymes
EA-B3A: ethacrynic acid
ERK: extracellular signal-regulated kinase
ERRα: ER-related receptor-α
FKBP12: FK506-binding protein
FRS2α: fibroblast growth factor receptor substrate 2
GFP: green fluorescent protein
GR: glucocorticoid receptor
GS: glutamine synthetase
GST: glutathione *S*-transferase
HECT: homologous to the E6-AP carboxyl terminus
HIF1α: hypoxia-inducible factor 1α
HMC: human mast cell
IAP: inhibitor of apoptosis protein
IFN: interferon
IRF4: interferon regulatory factor 4
MDS: myelodysplastic syndrome
MEIS2: myeloid ecotropic viral integration site 1 homolog 2
MM: multiple myeloma
NGF: nerve growth factor
PI3K: phosphatidylinositol-3-kinase
PROTACs: PROteolysis TArgeting Chimeras
RING: really interesting new gene
SARM: selective androgen receptor modulator
SMAD3: SMAD family member 3
SNIPERs: specific and non-genetic IAP-dependent protein erasers
TACC3: transforming acidic coiled-coil-3
TBD: thalidomide-binding domain
TrkA: tropomyosin receptor kinase A
VBC: VHL/Elongin B,C/CUL2 protein complex
VEGF: vascular endothelial growth factor
VHL: von Hippel–Lindau.
